# The discovery of a fossil whitefly from Lower Lusatia (Germany) presents a challenge to current ideas about Baltic amber

**DOI:** 10.1038/s41598-024-74197-8

**Published:** 2024-10-04

**Authors:** Jowita Drohojowska, Marzena Zmarzły, Jacek Szwedo

**Affiliations:** 1 Institute of Biology, Biotechnology and Environmental Protection, University of Silesia, 9, Bankowa St., Katowice, 40-007 Poland; 2 Laboratory of Evolutionary Entomology and Museum of Amber Inclusions, Department of Invertebrate Zoology and Parasitology, University of Gdańsk, 59, Wita Stwosza St., Gdańsk, 80-308 Poland

**Keywords:** Zoology, Palaeoecology, Geology, Palaeontology, Sedimentology, Evolution, Palaeontology, Taxonomy

## Abstract

The whiteflies (Hemiptera: Aleyrodidae) are small sternorrhynchan insects, which have the potential to cause significant economic damage to agricultural crops. There is a paucity of knowledge regarding the diversity, disparity, and evolutionary history of these insects, with classification based on the immatures, called puparia. The fossil record of whiteflies is sparse and incomplete, with the majority of fossils representing imaginal forms preserved as inclusions in fossilized resins. In this study, we present the first inclusion in succinite associated with the layers of Lower/Middle Miocene 2^nd^ Lusatian Lignite Seam of Wanninchen in Brandenburg, Germany. The objective of the present study is to elaborate this fossil, and as a consequence, a new fossil genus and species, *Pudrica christianottoi  ***gen. et sp. nov.**, is described. This fossil is a representative of the subfamily Aleyrodinae, and it is the third fossil genus of this whitefly subfamily to be described. The discovery of the fossil inclusion in the succinite from the lignite deposits of Lower Lusatia challenges the current understanding of the character and conditions of formation and deposition of central and east European Paleogene fossil resins. Succinite is a fossil resin that occurs in the Eocene deposits of the Gulf of Gdańsk, belonging to the Prussian Formation, containing a glauconite-rich horizon known as the ‘Blue Earth’. Similarly, glauconite-rich deposits are present in the Lublin area of Poland, where they are associated with the occurrence of succinite. Additionally, succinite has been found in deposits in the Rovno-Zhitomir area of Ukraine, which are alluvial deposits containing glauconite and lignite layers. Succinite was also identified in Eocene strata of Spitsbergen and in Axel Heiberg Island in the Canadian Arctic. Succinite has also been discovered in early Miocene deposits in Bitterfeld, Germany, where it occurs alongside lignite deposits (the deposit actually encompasses different fossil resins, so potentially originating from different source plants). Furthermore, it has been identified in younger (Pleistocene) deposits across Europe. The autochthonous (parautochthonous) character of the lignite deposits in Lower Lusatia raises questions regarding the time range of the succinite-producing gymnosperm trees and the autochthonous or allochthonous character of the lignite layers associated fossilized resins.

## Introduction

The Sternorrhyncha (Insecta: Hemiptera), comprising approximately 19,000 described recent species, represents a significant group of insects with both ecological and economic importance^1,2^. This suborder encompasses the infraorders Aphidomorpha (aphids, adelgids and phyloxerans), Coccidomorpha (scale insects), Aleyrodomorpha (whiteflies), and Psylloidea (psyllids) and a few extinct groups, viz. infraorders Pincombeomorpha, Naibiomorpha and Dinglomorpha, superfamily Protopsyllidioidea, family Liadopsyllidae^3,4^. The fossil record of Sternorrhyncha appears to date back to the Permian, with their evolutionary history extending to the Carboniferous^3^. However, the initial stages of their evolutionary development and diversification remain poorly understood. The prevailing view is that the Sternorrhyncha constitute a monophyletic lineage^3,5,6^. The sedentary lifestyles and phloem-feeding behaviors of these insects, which act as plant parasites, have resulted in a series of morphological reductions and losses, neotenous females, extreme sexual dimorphism, neometaboly and convergently derived morphological characters, as well as diverse endosymbiotic relationships. These factors would otherwise be useful in phylogenetic analyses^1,2^. As a result, establishing the relationships of Sternorrhyncha represents a significant challenge.

The Aleyrodidae, a sole family of Aleyrodomorpha, are commonly referred to as whiteflies. This nomenclature is derived from the presence of a powdery secretion, which is preened over the bodies and wings of the adults of almost all species. The majority of whiteflies are closely related to specific host plants^7,8^. If the number of described species is to be taken as an accurate guide, then whiteflies are by far the least speciose of the four extant groups of sternorrhynchans, with approximately 1,550 currently valid recent species^8^. The Aleyrodidae are currently subdivided into four subfamilies: the extinct Bernaeinae Shcherbakov, 2000 and the recent Aleyrodinae Westwood, 1840, the Aleurodicinae Quaintance et Baker, 1913 and the Udamoselinae Enderlein, 1909, the latter of which is of questionable taxonomic status^8^. The fossil record of the whiteflies is known to extend to the Late Jurassic period^9,10^. The occurrence of whiteflies preserved as fossils is relatively uncommon, a small number of fossils have been reported from sedimentary deposits and fossil resins, which have been dated to the Lower Cretaceous, Upper Cretaceous, Paleogene, and Neogene periods^9–22^.

### Geological setting

**Fig. 1:**
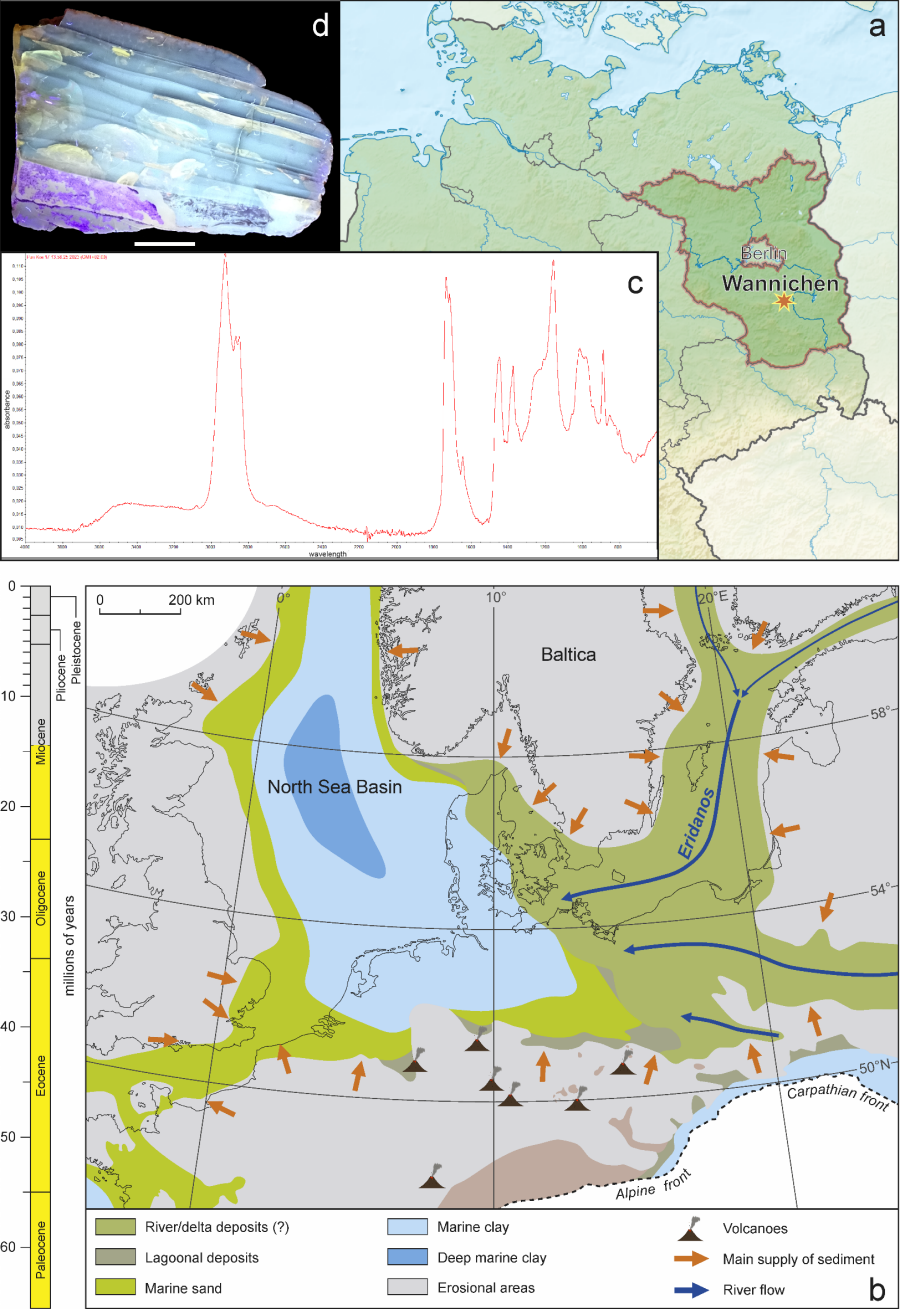
(a–d) Locality of succinite finding in the Brandenburg, Germany, modified after Alexrk2, CC BY-SA 3.0 (a), Middle Miocene (latest Langhian) palaeogeography, modified after Gibbard and Lewin 2016 (b), FT-IR spectrum of the resin sample (succinite) ATR and baseline corrected (c), UV light 395 nm image of the resin sample with layers and internal fractures visible, scale bar 0.5 mm (d).

The piece of succinite with inclusion described below was found in association with lignite chunks. The classic Lusatian lignite (brown coal)-mining district, located east of Elbe River, in the southern part of the federal states of Brandenburg and the eastern part of Saxony, is related to the North German-Polish Basin^23^(Fig. 1b). Brown coal has been known from the Lusatia area for more than 200 years. Accidental finds of ‘underground wood’ in the area around Zittau (Upper Lusatia) led to the first mining of lignite as early as 1740. Since 1864 there has been a lignite mine in the Senftenberg area and since 1856 in the Zeißholz area^24,25^. Most of the Miocene brown coal exploited in the coal-mining district of Lusatia (Lausitz) is part of the Cenozoic infill of the North German-Polish Basin^26^. The lignite-bearing sediments in the area result from several transgressive pulses with intercalated regressive phases^24,27^ The sea retreated at the beginning of the Paleogene, when the entire Bohemian Massif was influenced by tectonic, compressional stresses related with formation of Alpine-Carpathian fold belt in the south^28^. It was the time when dense, complicated network of old fractures and dislocations cutting Cadomian and Variscan basement were rejuvenated^29^. The Lusatian lignite developed around 12 to 17 million years ago from subtropical bog forests on lignite-free Oligocene layers^23,30^. The up to 250 m thick Neogene sediments of the Lusatian Coal District have been divided into sequences and layers based on complex lithostratigraphic modelling^27,31^. After the mining of the First Lusatian Seam, increasing open-cast mining in the 20th century focused on the deeper, 7–20 m thick Second Lusatian Seam. Basin-wide sequence-stratigraphic interpretation of Miocene sedimentary successions indicates that the Second Lusatian Seam was deposited during the TB2.2 (ca. 17.3–16.4 Ma; Late Burdigalian) and TB2.3 (ca. 16.4–14.8 Ma; Langhian) third-order cycles *sensu*^32^, implying that the Second Seam straddles the boundary between the Lower and Middle Miocene^26,33^. During the subsequent Ice Age these deposits were covered by glacial till of advancing glaciers and sediments originating from melting processes. The thickness of this cover layer varies from about 10 to 150 m.

The amber specimen comes from the layers in Wanninchen (Fig. 1a, b), near a post-mining lake Schalbendorfer See (the area of the town of Luckau, the municipality of Heideblick, district of Dahme-Spreewald, in the south of the federal state of Brandenburg). Schlabendorf-Süd open-cast lignite mine was named after the Schlabendorf district on the lake. The former Lübbenau and Vetschau power plants were supplied with raw coal from this open-cast mine between 1976 and 1991. Development of the open-cast mine began in 1975 and the first coal was extracted a year later. The open-cast mine was closed in 1991 and Lake Stiebsdorf and the Wanninchen and Drehnaer Weinberg and Lake Stiebsdorf nature reserves were created as post-mining landscapes, as well as the Marina Schlabendorf recreation and leisure centre on the lake^34,35^.

## Results

### Systematic palaeontology

Order Hemiptera Linnaeus, 1758.

Suborder Sternorrhyncha Amyot et Audinet-Serville, 1843.

Infraorder Aleyrodomorpha Chou, 1963.

Family Aleyrodidae Westwood, 1840.

Subfamily Aleyrodinae Westwood, 1840.

**Genus  *Pudrica * Drohojowska et Szwedo**,** gen. nov.**

Type species. *Pudrica christianottoi* sp. nov., here designated.

urn: lsid: zoobank.org: act: A2405912-00F7-421D-8C56-14F3B8B5DBDB.

**Etymology.** Generic name is derived from Lower Sorbian word ‘pudrica’ [pudrʲit͡sa] meaning a whitefly^36^. Gender: feminine.

**Diagnosis.** In general features and venation pattern similar to *Snotra* Drohojowska et Szwedo, 2016, with antennae 7-segmented, with antennomeres ratio F3:F4 = 2.0 (as in *S. christelae*, but ratio F3:F4 = 3.3; in *S. herczeki* antennae 6-segmented); antennomeres F3^d^ and F5^h^ with subapical rhinaria; compound eyes divided, connected by four ommatidia (compound eyes not dived in *Snotra*); thorax with mesopostnotum slightly concave in median portion, lateral sections convexly declivous laterad; forewing 2.3 times as long as wide (as in *S. herczeki*; in *S. christelae* 2.5); vein Sc + R almost straight, not forked (Sc + R curved in apical half, not forked in *S. herczeki*; vein Sc + R bent in apical half, weakly forked into Sc + Rs in *S. christelae*); vein CuA with more visible stem, then faint to obsolete (CuA not visible in *Snotra* and in other genera of the subfamily); CuP (claval fold) distinct, with apex reaching half of fore wing length (not reaching half of fore wing length in *Snotra*).

***Pudrica christianottoi ***sp. nov.,** Drohojowska et Szwedo**.

urn: lsid: zoobank.org: act:0DE167A6-8FE5-444D-96ED-FB95387746BA.

Figure 2a–h.

**Fig. 2:**
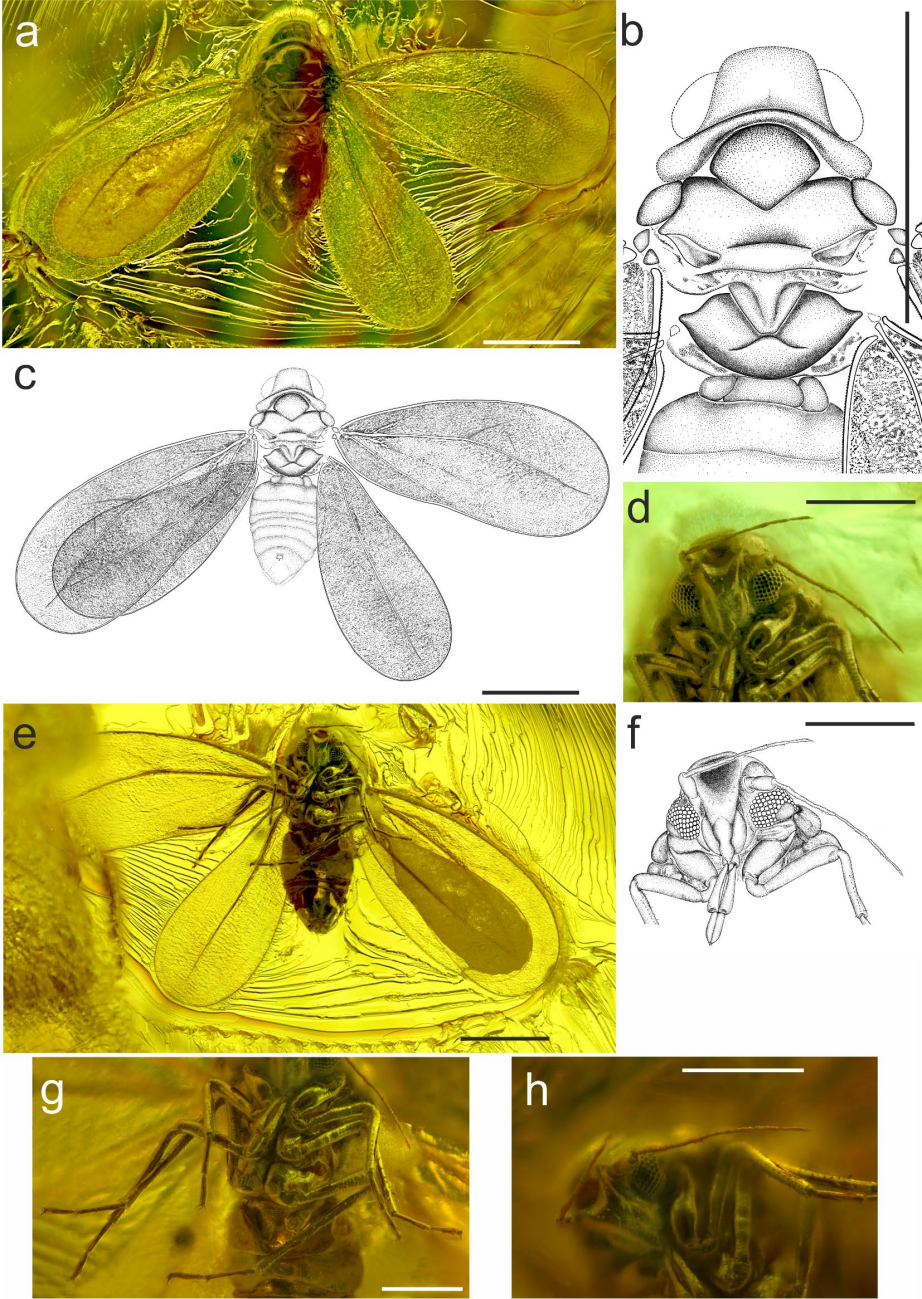
(a‒h) *Pudrica christianottoi  ***gen. et sp. nov.** imago. Body in dorsal view (a), Drawing of thorax (b), Drawing of body in dorsal view (c), Head in ventral side (d), Body in ventral view (e), Drawing of head in ventral side (f), Legs (g), Antenna (h); Scale bars: 0.5 mm (a, b,c, d,e, f); 0.25 mm (g, h). Drawings by Marzena Zmarzły.

**Diagnosis.** As for genus as it is the only included species so far.

**Description.** Measurements are given in Table 1. *Head* with compound eyes 0.88 times as pronotum, head in dorsal view trapezoid, anterior margin merely convex, lateral margins merely sinuately diverging posteriad, posterior margin concave, disc of vertex concave. Frons triangular, concave in upper portion, with lateral margins distinct, abruptly shifting to lateral areas of head capsule, clypeal suture arcuate, postclypeus subtriangular, narrowing ventrad to level of anteclypeus, convex, anteclypeus subpentagonal, wider in lower portion than tip of postclypeus, clypellus small, triangular; loral plated elongated, with upper angles exceeding level of clypeal suture, distinctly convex, lower angles reaching about half of anteclypeus median length. Apical segment of rostrum shorter than subapical one. Median ocellus absent, lateral ocelli distinct above the compound eyes, at level of anterior line of compound eye. Compound eyes in dorsal view flattened, not bulging, divided, connected in median portion with 4 ommatidia, ommatidia of equal size. *Antennae*: 7 antennomeres, antennal fovea at level of half of frontal height, scapus short, about 1/2 as long as pedicel, pedicel twice as long as wide, antennomere F3 twice as long as pedicel, antennomeres F4 and F6 of same length, half as long as antennomere F3, antennomere F5 slightly shorter than antennomeres F4 and F6, antennomere F7 longer than F6 antennomere with apical constriction (concavity) and single apical seta; subapical rhinaria visible on antennomeres F3 and F5; antennomeres F3, F5 and F6 with subapical setae, antennomere F7 with apical seta.

**Table 1:** *Pudrica christianottoi  ***gen. et sp. nov.** measurements (in mm).

	mm		mm
Body length total	1.15	Mesopostnotum width	-
Head with compound eyes width	0.31	Mesopostnotum length	-
Rostrum length 1^st^ part/ 2^nd^ part	0.22	Metascutum width	0.13
Pedicel length	0.03	Metascutum length	-
Scapus length	0.11	Metascutellum width	0.3
Antennomere F3 length	0.2	Metascutellum length	0.05
Antennomere F4 length	0.1	Profemur length	0.37
Antennomere F5 length	0.08	Protibia length	0.33
Antennomere F6 length	0.1	Basiprotarsomere length	0.13
Antennomere F7 length	0.12	Apical protarsomere length	0.1
Forewing length	1.46	Mesofemur length	0.25
Forewing width	0.64	Mesotibia length	0.37
Hind wing length	1.24	Basimesotarsomere length	0.13
Hind wing width	0.54	Apical mesotarsomere length	0.1
Pronotum width	0.35	Metafemur length	0.3
Pronotum length in mid line	0.03	Metatibia length	0.5
Mesopraescutum width	0.17	Basimetatarsomere length	0.17
Mesopraescutum length in mid line	0.14	Apical metatarsomere length	0.13
Mesoscutum width	0.35	Abdomen length including genitalia	0.6
Mesoscutum length in mid line	0.05		
Mesoscutellum width	0.17		
Mesoscutellum length	0.05		

*Thorax*. Pronotum arcuate, anterior margin arcuate, diverging posteriad, lateral margins broader than length of pronotum in mid-line, with lateral margins rounded, posterior margin deeply arcuate. Mesopraescutum 1.2 times as long as broad, anterior margin arcuate, posteriad margins converging, straight, apex slightly rounded, with angle slightly wider than rectangular angle. Mesoscutum about as wide as pronotum, mesoscutum 6 times as wide as long in mid-line; anterior margin triangularly concave in median portion, lateral sections straight, anterolateral margin angulately rounded, lateral margin concave, diverging posteriad, lateral angles acute, posterolateral margins slightly sigmoid, posterior margin slightly concave; parapteron large, ellipsoid, flattened; mesoscutellum in form of narrow band, 3.4 times as wide as long in mid-line, anterior margin arcuate, posterior margin arcuately convex; mesoscutellum and mesopostnotum with a break due to deformation; mesopostnotum subtriangular, with median shallow concavity, lateral portions convexly declivous laterad; lateral bands of mesoscutellum and mesopostnotum visible; metascutum with distinct median incision, deeper than half of its length in mid-line, lateroanterior margins distinctly sinuate, anterior angles widely arcuate, lateral angles distinctly elongately acute, posterolateral margin arcuate, posterior margin convex; metascutellum widely ellipsoid, about 3 times as wide as long in mid-line, anterior margin convex, posterior margin convex, lateral angles acute.

Both pairs of wings preserved in very good condition.

*Fore wing* 2.3 times as long as wide, widest at 2/2 of fore wing length, widening apicad, costal margin merely arcuate, anteroapical angle widely rounded, apex arcuate, posteroapical angle arcuate, tornus (postclaval margin) widely arcuate, claval margin arcuate, with claval angle widely angulate; clavus well separated, with claval old (CuP) well visible, weakened in very apical portion, slightly prolonged on membrane, apex of clavus reaching 1/2 of fore wing length; margin of fore wing with small tubercles and microsetae; stem Sc + R straight at base, bent at half of fore wing length, at wide angle (ca. 140°), not reaching the margin, stem CuA leaving common stem at about 15°, more distinct in basal section, then weakened to obsolete, directed to posteroapical angle not reaching the margin; vein CuP distinct, weakened to faded in apical portion merely prolonged to membrane; very weak, slightly traceable vein Pcu on clavus; corium and clavus with distinct, irregular sculpturing, membrane with more regular sculpturing, a short fold visible at base of Sc + R, another short and weak fold (remnant of ScP + R branch?) at level of Sc + R bending.

*Hind wing* shorter than fore wing, ca. 0.85 of fore wing length, 2.3 times as long as wide. Costal margin bent at base, then straight, merely concave in at basal 1/4 of hind wing length, then widely arcuate, anteroapical angle widely arcuate, apex rounded, posteroapical angle widely arcuate, posterior margin arcuate; margins covered with granules; two macrosetae interspersed with microsetae at the base, three macrosetae interspersed with microsetae at apical 1/2 of hind wing; vein R slightly sigmoid, arcuate at base, then merely arcuate, directed to the hind wing apex, but not reaching the margin; hind wing with distinct irregular sculpturing.

*Legs* well visible; metaleg the longest, mesoleg appears the shortest (skewed view), profemur and mesotibia the same length. Tarsi 2-segmented, basitarsomere about 1.3× longer than apical tarsomere of pro-, meso- and metaleg, basiprotarsomere and basimesotarsomere the same length, apical protarsomere the same length as apical mesotarsomere, paronychium thin, finger-like, almost the same length as claws, tibial combs well visible, composed of several long and relatively even distributed setae.

*Abdomen* from the dorsal side abdomen seems quite massive, strongly narrowed at the thorax. Genitalia the same length as the total length of the head and thorax. Wax plates invisible. Gender cannot be determined. Probably female due to characteristic triangular shape of apical part of the abdomen and the lack of visible claspers or aedeagus.

**Holotype.** Imago, sex not to be recognized (probably female), GPIH 5083 deposited in Leibniz-Institut zur Analyse des Biodiversitätswandels, Museum der Natur Hamburg-Geologie, Hamburg, Germany. Inclusion in succinite from deposits of former Schlabendorf-Süd opencast mine (Fig. 1a, b). *Condition of the specimen.* A small piece of amber measuring 2.5 cm × 0.8 cm × 0.5 cm, approximately trapezoidal in shape. Layered succinite (Fig. 1c, d), inclusion of whitefly preserved in good condition, both sides are visible, placed on the border between the layers. The piece of amber with syninclusions—stellate hairs, bubbles of gas, and internal fractures (scale-like), no traces of strong weathering or sever alteration.

**Etymology.** Specific epithet is given after founder of the specimen Mr. Christian Otto.

**Age and occurrence.** Succinite of probably Middle-Late Eocene age (?), most probably redeposited, found in association with lignite and glessite, in layers without gravel and pebbles (typical for Pleistocene strata), most probably representing Lower/Middle Miocene, Burdigalian/Langhian; Brieske Formation, Welzow Member, 2^nd^ MFH (2. Miozänen Flözhorizontes; 2^nd^ Miocene Seam Horizon), Wanninchen ad Schlabendorfer See, municipality of Heideblick, district of Dahme-Spreewald, Brandenburg / Waninki ad Chóžyšćański jazor, Heideblick, wokrejs Damna-Błota, Bramborska^24,33,37^.

## Discussion

Fossil resins have been identified in the Miocene lignite deposit and in reworked layers of the Quaternary age^38–42^. The identified fossil resins from deposits in Lusatia comprise glessite and succinite^38^, as well as a resin similar to siegburgite^41,43^. Additionally, there are unidentified resins^42^. The inclusions in fossil resin were documented by Sauer^38^ and Stiegler & Stiegler^39^. These inclusions included beetles, flies, ants, moths, true bugs, planthoppers, termites, plecopterans, and collembolans. However, there is a lack of formal descriptions or definitive identifications. The aforementioned fossil represents the inaugural formally named fossil from this deposit, as well as the first fossil whitefly documented from Lower Lusatia strata.

The whitefly subfamily Aleyrodinae comprises approximately 1550 species distributed across over 140 described genera, yet the precise relationships between these groups remain uncertain. A subdivision into 13 tribes has been proposed based on puparia^44^. However, due to the current state of whitefly systematics, they have been largely underutilised. A significant proportion of the distinguished genera are represented by only one or two species, and their relationships remain poorly understood. Before a robust classification of tribes and their relationships can be proposed, there is a need for extensive research on a global scale. Fossil record of Aleyrodinae is scarce, comprising the genus *Baetylus* from Lower Cretaceous, Barremian, amber of Lebanon^15^ and two species of the genus *Snotra* from the Eocene succinite from Gulf of Gdańsk deposit^45,46^. In the Barremian *Baetylus* antennae comprise 10 antennomeres, antennomeres ratio F3:F4 = 0.98, fore wings much more elongated, 2.8 times as long as wide. In the Eocene *Snotra christelae* antennae are 7-segmented with antennomeres ratio F3:F4 = 3.3, fore wing 2.5 times as long as wide. Interestingly, in *S. herczeki* some antennomeres seems to be fused and antenna consist of 6 antennomeres, while in vast majority recent taxa, e.g. in *Aleyrodes* spp. antenna usually consist of 7 antennomeres^15^. *Pudrica  ***gen. nov.** presents antennae with 7 antennomeres, with ratio F3:F4 = 2.0, fore wing 2.3 times as long as wide. A feature of importance as a distinguishing character is the number of ommatidia connecting upper and lower lobe of compound eyes. In the ancient forms of whiteflies, including *Baetylus*, compound eyes are not divided, in *Snotra* compound eye is reniform with posteroventral portion composed of smaller ommatidia. In *Pudrica  ***gen. nov.** compound eye is divided, connected in median portion with 4 ommatidia, and the ommatidia are of equal size. In males of extant aleyrodicine whiteflies upper and lower portions of the compound eyes can be connected with one ommatidium and/or with several ommatidia to completely separated^7,47^. This character however, appears underexplored, as most of morphological features in imagines of extant whiteflies, and it needs more material from different genera to be examined, to estimate its phylogenetic importance. The same situation is e.g. with patterns observed in mesotibial brushes and metatibial combs and brushes^48^, the features not always available in the fossil material. Schlee^11^ concluded that the Aleyrodinae was the first to achieve survival success by feeding on gymnosperms, with a broader host range, but generally preferring dicotyledones, especially fabaceaous hosts over the others^49^. However, relationships between whiteflies and their host plants remains unclear^44,50^. Analysis provided by Manzari & Quicke^44^ revealed that aleurodicines are reported from the two advanced groups, i.e. asterids (including euasterids I and euasterids II) and rosids (including eurosids I and eurosids II), two of the most recently derived groups according to APG IV flowering plant classification system^51^.

The lignite deposits exploited in Schlabendorf-Süd mine were formed in swamp and bog environments, under more or less marine influence. The light micaceous sands deposited in shore face or barrier island environments; the silts representing lagoon environments and in part tidal flats as weil^27,37,52^. The deposits were formed during the Middle-Miocene Climatic Optimum, and palaeobotanical data suggested various types of mixed mesophytic forests, forest- to bush moors to reed-mire vegetation, with the predominance of subtropical taxa^53–55^. Lignite deposits with fossilized wood and resins appear to be autochthonous, with gymnosperm plants from families Cupressaceae, Sciadopityaceae and Pinaceae identified (succinite postulated mother-plants). These plants may be the source of part of the fossilised resins. The discovery of glessite, derived from the angiosperm Burseraceae (*Canarium* Linné, 1754) and resin comparable to siegburgite (derived from *Liquidambar* Linné, 1753; Altingiaceae), provides further evidence of the diverse range of fossilised resins, thus reinforcing their autochthonous nature. It is, however, not possible to rule out the possibility that at least some of the resins may have migrated to the Miocene layers at a later date, as suggested by Wolfe et al.^56^ in relation to deposits in the Bitterfeld area. The allochthonous character of these resins can be supported by the existence of a wide plain before the formation of the Baltic Sea, which was occupied by a substantial river termed the ‘Eridanos’ or ‘Baltic’ river system during the Miocene^57^ (Fig. 1b), which denuded the resins from older deposits.

## Conclusions

The discovery of Aleyrodine whitefly in succinite from Lower/Middle Miocene sediments provides new insights into the taxonomic diversity and morphological disparity of these insects. This is the first discovery of these insects in Miocene sediments (but in resin of probably older age – Middle-Late Eocene (?)); however, a more substantial corpus of data and their interpretation are necessary to either corroborate or refute the postulated age of the inclusions. Our results emphasise the necessity of considering a comprehensive range of data, encompassing both geological and biological information, in order to gain insight into the evolutionary processes that shaped the morphology and palaeoecological interactions of the fossil. The fossil aleyrodine whitefly described above may challenge existing hypotheses regarding the timing and conditions of the formation and deposition of fossilised resins in Europe.

## Materials and methods

The specimen was examined, photographed and measured using the Leica M205C, Nikon SMZ25, Nikon SMZ1500, Nikon SMZ1270, Nikon Eclipse E600 digital microscopes platforms, with incident and transmitted light were used simultaneously as well as with fluorescent illumination. The illustrations were prepared with two image-editing software packages (CorelDraw X9, CorelPaintX9). Fourier Transform Infrared Spectra was obtained in the Amber Experts company laboratory, Gdańsk with Thermo Scientific Nicolet 380 FT-IR Spectrometer, with ATR and baseline correction, for the reasons and according to procedure proposed by Szwedo & Stroiński^58^. The received spectrum was compared with data provided in Kosmowska-Ceranowicz^59^. Morphological terminology after Drohojowska & Szwedo^15^ and Gerling^7^. This paper is registered in ZooBank under LSID: urn: lsid: zoobank.org: pub:14ACE0B4-2918-4B41-99BD-DA24361B44D1.

## Data Availability

All data generated or analysed during this study are included in this published article.
